# Unveiling the Impact of Rapeseed Meal on Feeding Behavior and Anorexigenic Endocrine in *Litopenaeus vannamei*

**DOI:** 10.3390/ani14040540

**Published:** 2024-02-06

**Authors:** Bo Zhou, Hongmei Ran, Qijun Zhang, Hu Chen, Fenglu Han, Chang Xu, Qun Zhao

**Affiliations:** 1 Fisheries Research Institute of Sichuan Academy of Agricultural Sciences, Yibin 644000, China; zhoubo2008@163.com; 2 Key Laboratory of Tropical Hydrobiology and Biotechnology of Hainan Province, Hainan Aquaculture Breeding Engineering Research Center, School of Marine Biology and Fisheries, School of Breeding and Multiplication (Sanya Institute of Breeding and Multiplication), Hainan University, Haikou 570228, Chinaflhan@hainanu.edu.cn (F.H.); cxu@hainanu.edu.cn (C.X.); zhaoq@hainanu.edu.cn (Q.Z.)

**Keywords:** white leg shrimp, aquaculture, fish meal substitution, rapeseed meal, appetite, neuropeptides

## Abstract

**Simple Summary:**

Discovering alternatives to fish meal is crucial for maintaining the stability and rapid development of aquaculture. Rapeseed meal stands out as a promising substitute due to its low price, renewability, high protein content, and high yield. However, there are still several challenges associated with the use of rapeseed meal in aquatic feeds, one of which is anorexia. Rapeseed meal-induced anorexia has been reported in numerous fish studies, but its impact on crustacean feeding remains unelucidated. This study investigated whether rapeseed meal could cause anorexia in *Litopenaeus vannamei*, which is a crustacean that can accept a large amount of plant protein and is the largest cultured crustacean in the world. Considering the key role of neuropeptides in anorexia, transcriptome analysis was performed to identify six neuropeptides directly involved in rapeseed meal-induced anorexia. This study confirmed that rapeseed meal causes anorexia in *L. vannamei*, and the finding can be used to improve the application of rapeseed meal in shrimp feed. In addition, the neuropeptide response to rapeseed meal-induced anorexia in shrimp was explored, providing a theoretical basis for reducing rapeseed meal-induced anorexia via the endocrine pathway.

**Abstract:**

*Litopenaeus vannamei*, with high plant protein acceptance and high global aquaculture production, is a potential species for rapeseed meal application. However, rapeseed meal has been associated with anorexia in fish, and whether the same occurs in *L. vannamei* remains unknown. This study demonstrated the effects of rapeseed meal on the feeding and anorexigenic endocrine of *L. vannamei* based on feeding behavior and transcriptomics. Soybean meal was replaced with fermented rapeseed meal (50%), and a significant increase in remaining diet and dietary discard was observed with a significant reduction in dietary visits. Transcriptome analysis revealed that the pathways involved in rapeseed meal-induced anorexia mainly included signal transduction, the digestive system, the sensory system, the endocrine system, phototransduction–fly, the thyroid hormone signaling pathway and pancreatic secretion. Moreover, this study further analyzed and identified seven neuropeptides involved in rapeseed meal-induced anorexia, and it explored the complex expression regulation strategies of these neuropeptides. In summary, this study confirmed through feeding behavior that rapeseed meal causes anorexia in *L. vannamei*, and it identified seven neuropeptides that were closely related to the anorexia process.

## 1. Introduction

Oil-seed meals are cheap, renewable, have a high protein content, and are widely used in aquatic feeds to replace fish meal; Therefore, they play an essential role in the sustainable development of aquaculture [1]. Compared with other oil-seed meals, soybean meal has high nutritional value, a balance of essential amino acids, and few anti-nutrients, making it the most widely used and most effective fish meal substitute in aquatic feeds [2,3]. Due to the sharp increase in the demand for soybean meal in animal feed, its price has soared and is no longer as economical. According to Federal Reserve Economic Data, the price of soybean meal in 2022 is 2.5 times that of 2002, rising from about US$200 per ton to nearly US$500 per ton (https://fred.stlouisfed.org/series/PSMEAUSDA, accessed on 25 December 2023). Therefore, developing applications for other oil-seed meals other than soybean meal may represent an attractive solution for aquaculture.

Rapeseed meal is considered a promising protein ingredient for aquafeeds due to its global production availability, low price, and balanced amino acid profile compared to other plant proteins. Rapeseed meal is a by-product of the oil industry and is the most abundant protein feed ingredient after soybean meal [4]. Based on rapeseed’s broader global planting potential, rapeseed’s production has grown much faster than soybean with a 97% increase from 2003 to 2013 compared with 45% for soybeans [4]. In addition, rapeseed meal has a higher content of sulfur-containing amino acids compared to soybeans, which contributes to the amino acid balance of formulations [5]. Furthermore, soybean protein is also used in human food, which limits its applications in aquafeeds [1]. Finally, rapeseed meal remained at a relatively stable price of US$200 per ton over the past 10 years, which has been extremely attractive to the aquatic feed industry (https://www.investing.com/commodities/rapeseed-meal, accessed on 25 December, 2023). However, the anti-nutrients in rapeseed meal such as glucosinolates, phytic acid, sinapinic acid, tannins and non-starch polysaccharides can lead to low utilization and adverse effects on production [5,6,7,8]. Rapeseed meal was found to reduce feed intake, which is one of the main obstacles to its application [6]. However, only a few studies have researched the regulatory effects of rapeseed meal on appetite.

Appetite is a complex and finely regulated system that involves a series of neuroendocrine regulation, in which neuropeptides play a key role in appetite control [9,10]. Research on appetite-regulating neuropeptides is mainly focused on vertebrates, while little research has been performed on crustaceans [11]. Existing studies confirmed that rapeseed meal suppresses appetite in many common aquaculture fish, including *Oncorhynchus mykiss* [12], *Ctenopharyngodon idella* [13], and *Sparus aurata* [14]. Furthermore, the neuropeptide research findings of vertebrates cannot be extrapolated to crustaceans due to their distinct nervous systems and neuroendocrine patterns [15,16]. The development of novel identification techniques for neuropeptides has enabled further research on crustaceans [17,18,19]. Mass spectrometry and transcriptome analysis have revealed the functions of crustacean neuropeptides in processes such as hypoxia [20,21], immunity [16] and reproduction [22]. In particular, the transcriptome, with characteristics of simple sample preparation, low cost, and easy data analysis, has gradually become the main method for the identification of neuropeptides in crustaceans [18,22,23]. Recently, studies have also focused on the neuropeptide regulation of crustacean feeding [23,24]. However, *Litopenaeus vannamei* is the crustacean with the largest aquaculture production in the world, and no research has focused on the appetite regulation mechanism of its neuropeptides.

According to the FAO’s 2020 forecast, the global annual production of *L. vannamei* will exceed 5 million tons (the output in 2020 is about 4.5 million tons) and may continue to maintain a Compound Annual Growth Rate (CAGR) of more than 5%. Since the demand for feed protein in *L. vannamei* is as high as 45%, solving the supply of feed protein sources is the key to maintaining the current status and ensuring the rapid growth of the shrimp aquaculture industry. Therefore, exploring new feed protein sources and solving existing problems in their application are the focus of current *L. vannamei* feed research. This study takes the globally farmed crustacean *L. vannamei* as the research object, focusing on the key issue of anorexia caused by rapeseed meal in feed formulations, and uses one of the best identification methods for neuropeptides—transcriptome—to explore rapeseed neuropeptide associated with anorexia. The study findings provide a theoretical basis for the application of rapeseed meal in *L. vannamei* feed.

## 2. Materials and Methods

### 2.1. Diet Design and Preparation

Previous studies have shown that replacing fish meal with 85% soybean meal (RSM0) does not affect production performance. Subsequently, rapeseed meal was used to replace 0% (RSM0), 25% (RSM25), 50% (RSM50), 75% (RSM75) and 100% (RSM100) of soybean meal, respectively. When the replacement ratio reached 50%(RSM50), it would lead to a significant decrease in the production performance of *L. vannamei*. Therefore, two feeds with equal nitrogen and equal lipids were prepared. The soybean meal group (RSM0) containing 150 g·kg^−1^ fish meal and 396 g·kg^−1^ soybean meal was used as the control group. On the basis of RSM feed, fermented rapeseed meal was used to replace soybean meal at a ratio of 50% (RSM50). Fish meal, shrimp shell meal, peanut meal, soybean meal and/or fermented rapeseed meal were used as dietary protein sources; cornstarch was used as a dietary carbohydrate source; soy lecithin, soybean oil and fish oil were used as the main lipid sources. In addition, methionine and lysine were supplemented in the diet to meet the nutritional needs of *L. vannamei*. The diets formula are displayed in Table 1.

The feed ingredients (fish meal, soybean meal, shrimp shell powder, peanut meal and fermented rapeseed meal) were crushed and passed through a 60-mesh sieve. The step-by-step expansion method was used to thoroughly mix all dry ingredients, and distilled water and oil were added to the mixture, which was evenly stirred in a mixer. After reaching the appropriate humidity, a twin-screw plodder (CD4 × 1TS, SCUT, Guangdong, China) was used to compact the mixture into feed pellets with a diameter of 1.0 mm. The pellets were ventilated and dried at 16 °C to a moisture content of ≤10% and then stored at −20 °C for subsequent experiments.

### 2.2. Aquaculture and Sample Processing

Twenty-four shrimps (9.3 ± 0.6 g) were selected and randomly assigned to two highly transparent glass fish tanks (90 cm × 60 cm × 50 cm) with 12 shrimps in each tank. Two fixed-position feeding trays were placed in each tank. Four days of mixed diet (RSM0:RSM50 = 1:1 by weight) feeding were performed to acclimate the shrimp to feed in the feeding tray and adapt to the experimental conditions. During this period, each feeding tray was fed four times a day (8:00, 12:00, 16:00 and 20:00), and 0.5 g of mixed feed was fed each time, which means that each tank was fed 4 g of mixed diet every day.

For the collection of feeding behavior assessment videos, the shrimps were fed four times a day according to the previous procedure, and the two feeding trays in one tank were randomly assigned RSM0 or RSM50 each feeding. No matter what kind of feed was allocated to the feeding tray, the amount of each feed was 0.5 g. Based on food intake and lighting requirements for video shooting, feeding at 16:00 was video recorded, and video shooting lasted 10 min after the meal. The same procedure was performed for video recording of feeding behavior in both tanks and was repeated for 3 consecutive days.

Considering the high correlation between feeding behavior and endocrine system and the fact that the shrimps in the two tanks were under the same procedure in the previous experiment, the shrimps were continued to be used in the study of the effects of rapeseed meal on the endocrine system. The shrimps were fed and adapted according to the previous adaptation program until before 16:00 on the fourth day. Then, at 16:00, the shrimps in one tank were fed an overdose (5 g) of RSM0 as the control group, and the shrimps in the other tank were fed an overdose (5 g) of RSM50 as the treatment group. At 16:30, the shrimps were all caught quickly and decapitated, and then the nervous system (eyestalks, brain, perifeeding ganglia, and abdominal ganglia) were dissected and collected within 30 min. The 12 samples obtained from each of RSM50 or RSM0 were randomly divided into 3 parallel groups, and each parallel group included a mixture of 4 samples. The collected samples were stored in animal tissue RNA preservation solution in a −80 °C refrigerator for later use. The water temperature was kept at 28 ± 2 °C throughout the experiment, and two-thirds of the water was replaced at 14:00 every day to maintain water quality. This study was approved by the Animal Welfare Ethics Committee of Hainan University, and the approval number is HNUAUCC-2023-00129.

### 2.3. Feeding Behavior Indicator Statistics

Dietary visits, feeding time, and dietary discarding were manually counted from the feeding videos. Dietary visits were counted as one when the shrimp arrived and completely left the feeding tray. Feeding time was the cumulative time spent by all shrimp visiting a feed tray. Diet discarding referred to the amount of dietary discarded after being taken away by the shrimp. The remaining diet weight after feeding was calculated using the feed particle-counting method. Briefly, the total weight of 100 pellets was weighed and used to calculate the average pellet weight. The remaining diet weight was calculated by multiplying the average particle weight by the number of remaining particles.

### 2.4. Transcriptome Analysis

The total RNA of the nervous system samples of the RSM0 and RSM50 groups was extracted using the Trizol method (Takara Co., Tokyo, Japan). The concentration, purity and integrity of RNA were assessed using Agilent 2100 (Aglilent, Santa Clara, CA, USA) and agarose gel electrophoresis. Subsequently, a TruSeq mRNA LT Sample Prep Kit (Illumina, San Diego, CA, USA) was used to prepare cDNA libraries. A Quant-iT PicoGreen dsDNA Assay Kit (Invitrogen, Carlsbad, CA, USA) was used to determine the total concentration of the library, and the library was diluted to 10 nmol/L and sequenced using NovaSeq 6000 (Illumina, USA). The raw data obtained by sequencing were filtered and quality controlled using Cutadapt (v2.7) software and FastQC (v0.11.8) software to obtain clean reads. HISAT2 (v2.1.0) was used to align clean reads to the *Litopenaeus vannamei* reference genome, and HTSeq software (v0.9.1) was used to count the number of clean reads aligned to each gene. The transcripts per kilobase of exon model per million mapped reads (TPMs) value of the transcript was calculated to normalize the expression level, and DESeq software (v1.39.0) was used to perform differential analysis of gene expression. TopGO (v2.40.0) was used for GO enrichment analysis, and Clusterprofiler software (v3.16.1) was used for KEGG enrichment analysis. Based on the progress in the identification of neuropeptides in crustaceans [23,25,26], a private neuropeptide database was constructed. An online sequence comparison tool (https://www.omicshare.com/tools/Home/Soft/blastcmp, accessed on 25 December 2023) was used to compare differentially expressed genes with the private neuropeptide database to screen for differentially expressed neuropeptides.

### 2.5. Statistics and Analysis

The data in this study were presented as mean ± standard error. GraphPad Prism 9.5.0 was used for statistical analysis. After the F test, a paired *t*-test was performed to compare the experimental and control groups. In this study, *p* < 0.05 was considered statistically significant and marked with *; *p* < 0.01 was considered statistically highly significant and marked with **; *p* < 0.001 was considered statistically extremely significant and marked with ***.

## 3. Results

### 3.1. Effects of Rapeseed Meal Replacing Soybean Meal on Appetite

**Figure 1:**
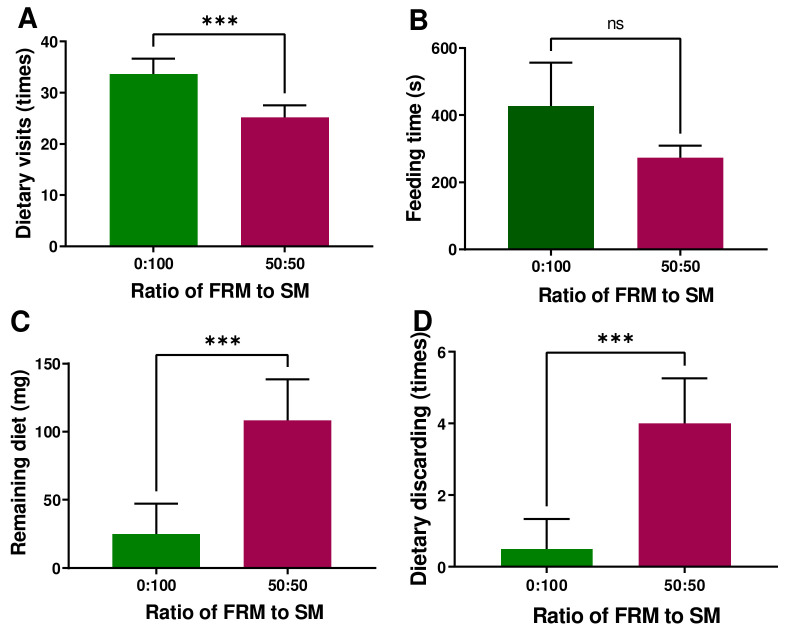
Effects of replacing soybean meal with rapeseed meal on dietary visits (**A**), feeding times (**B**), remaining diet (**C**), and dietary discarding (**D**). “***” indicates significant difference between the two groups and *p* < 0.001, “ns” indicates no significant difference between the two groups.

The appetite-reducing effects of rapeseed meal have been demonstrated by multiple lines of evidence, including the dietary visits, remaining diet, and dietary discarding (Figure 1). When 50% of soybean meal was replaced with fermented rapeseed meal, a significant decrease in dietary visit was observed (*p* < 0.001), whereas the remaining diet (*p* < 0.001) and the dietary discarding (*p* < 0.001) increased significantly (Figure 1). However, feeding times did not show a statistically significant difference (*p* = 0.1523), which might be attributed to high standard errors. The shorter feeding times due to rapeseed meal are reflected in Figure 1.

### 3.2. Differential Genes Response to Rapeseed Meal Diet

**Figure 2:**
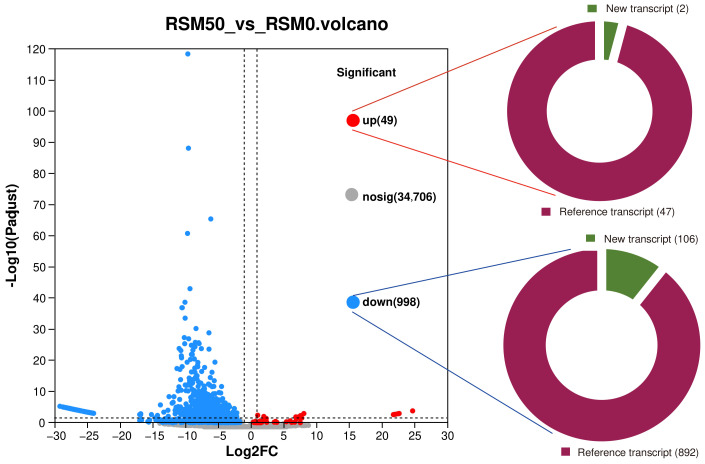
Differential genes response to rapeseed meal diet.

A total of 1047 differentially expressed genes were found in the nervous system of *L. vannamei* between the RSM0 and the RSM50 groups. Among the differential genes, 49 genes were up-regulated and 998 genes were down-regulated (Figure 2). Among the 49 up-regulated genes, 48 were reference transcripts and 2 were newly discovered transcripts. Among the 49 up-regulated genes, 892 were reference transcripts and 106 were newly discovered transcripts (Figure 2).

### 3.3. Annotation and Enrichment of Differential Genes in Response to Rapeseed Meal

**Figure 3:**
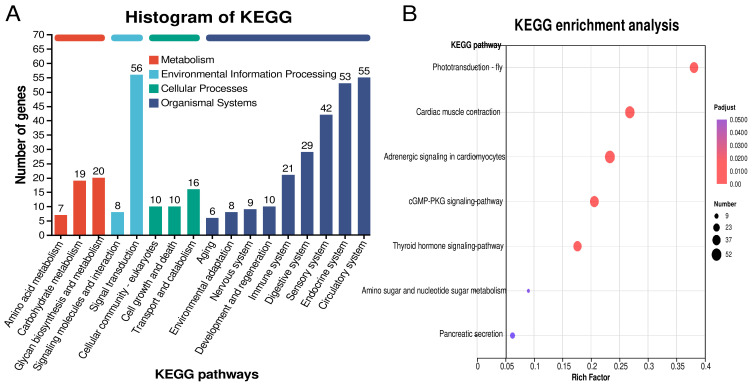
Functional annotation and enrichment of pathways in response to rapeseed meal diet.

The functional annotation and enrichment results of the differential genes showed that rapeseed meal mainly affected biological functions such as digestion and absorption, nutrient metabolism and rhythm. In terms of functional annotation, the KEGG pathways with more than 25 annotated genes include signal transduction (56 genes), digestive system (29 genes), sensory system (42 genes), endocrine system (53 genes) and circulatory system (55 genes) (Figure 3A). In KEGG functional enrichment, differential genes were enriched in a total of seven pathways. Among the pathways closely related to feeding were phototransduction–fly, the thyroid hormone signaling pathway, amino sugar and nucleotide sugar metabolism and pancreatic secretion. Interestingly, the other three pathways enriched by KEGG were all related to cardiovascular function and metabolism (Figure 3B).

### 3.4. Potential Neuropeptides in Response to Rapeseed Meal Diet

The private crustacean neuropeptide database generated in this study included a total of 430 neuropeptides. The database was compared with proteins encoded by differential transcripts, and 145 comparison results were obtained. The results indicated that 50 amino acid sequences of neuropeptides in response to rapeseed meal diets were encoded by 49 transcripts expressed from 18 loci. Among them, 15 newly discovered transcripts encode the amino acid sequences of 16 neuropeptides, and 34 reference transcripts encoded the amino acid sequences of 34 neuropeptides. The numbers of loci encoding 10, 5, 3, 2, and 1 transcripts were 2, 1, 3, 4, and 8, respectively (Supplementary Material File S1, Supplementary Table S1). Notably, multiple neuropeptide subtypes have small amino acid sequence differences, which results in the amino acid encoded by the same CDS being aligned by multiple neuropeptide subtypes (Supplementary Material File S1).

### 3.5. Neuropeptides in Response to Rapeseed Meal Diet

To eliminate the interference of similar neuropeptides being aligned to the same amino acid sequence, the alignment results with the highest consistency with the neuropeptide library, and expecting < 1, identities > 50% and positives > 50% were used for the next analysis. The obtained amino acid sequences encoding neuropeptides were derived from 15 transcripts expressed from six gene loci (Table 2). Among them, the neuropeptide identities and positives encoded by LOC113824994 and LOC113819780 were both 100% (Supplementary Table S1).

**Figure 4:**
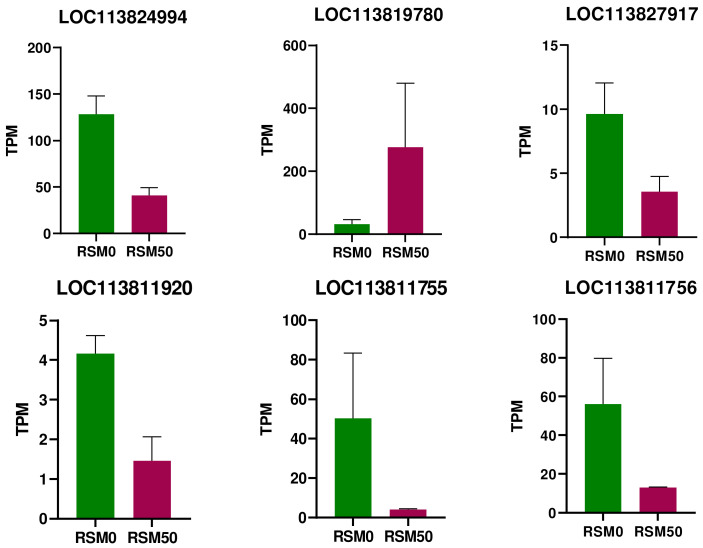
Expression of key neuropeptide genes in response to rapeseed meal-induced anorexia.

The expression levels of six gene loci in response to rapeseed meal-mediated anorexia in shrimp are presented in Figure 4. In terms of TPM expression, except for the significant increase in the expression of LOC113819780, the expression of the remaining five loci decreased, including LOC113824994, LOC113827917, LOC113811920, LOC113811755, and LOC113811756 (Figure 4).

**Figure 5:**
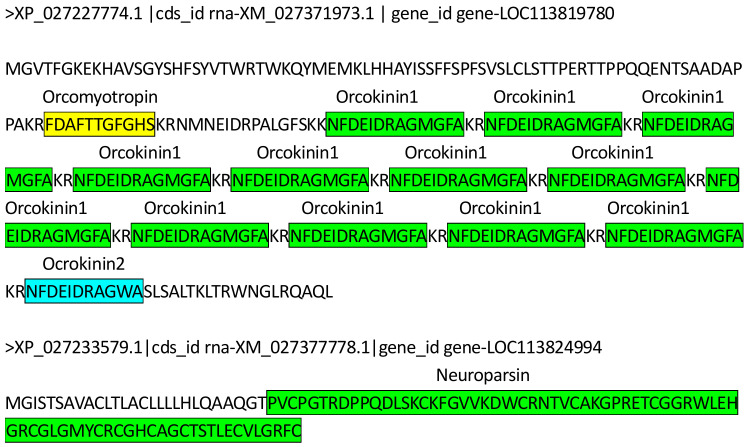
Proteins and neuropeptides encoded by LOC1138249494 and LOC113819780. Different neuropeptides are marked with different colored boxes.

Furthermore, the expression of the neuropeptides defined in this study involves a complex secretion process. The LOC113819780 locus encodes only one protein, which is produced into three types, a total of 14 neuropeptides, including 1 orcomyotropin, 12 orcokinin1, and 1 orcokinin2 (Table 2, Figure 5). In addition, the neuropeptide YRamide precursor-related peptide (YRPRP4) expressed nine transcripts from two loci, of which LOC113811755 produced eight transcripts (Table 2).

## 4. Discussion

Rapeseed meal is a potential aquatic feed protein source that may be used to supplement soybean meal. However, its application in aquaculture is limited due to causing anorexia. This study confirmed through observation of feeding behavior that rapeseed meal caused anorexia in *L. vannamei*. Considering the important role of neuropeptides in the regulation of anorexigenic responses, the transcriptome, a commonly used discovery method for neuropeptides, was used to explore the neuropeptide response pathways of rapeseed meal-induced anorexia in *L. vannamei*. This study discovered that seven neuropeptides encoded by six transcripts may be involved in the anorexigenic regulation of *L. vannamei* caused by rapeseed meal.

As the potential application of rapeseed meal in aquatic feed is being explored, a growing number of studies have reported that rapeseed meal causes anorexia in aquatic animals. The addition of rapeseed meal to the diet has induced anorectic responses in aquatic animals such as *Oncorhynchus mykiss* [12], *Ctenopharyngodon idella* [13], *Sparus aurata* [14], *Pelteobagrus ussuriensis* [27], *Rachycentron canadum* [28] and *Psetta maxima* [29]. Kaiser et al. reviewed the components of rapeseed meal that were responsible for inducing anorexia in aquatic animals, which included glucosinolates, phytic acid, non-starch polysaccharides, tannins, sinapines, amino acid imbalance, etc. [5]. The above evidence highlighted that rapeseed meal has an extremely widespread anorectic effect on aquatic animals, whether they are carnivorous species or omnivorous species, whether they are freshwater species or seawater species. Unfortunately, in crustaceans, another major department of aquaculture, few studies have focused on the effects of rapeseed meal on crustacean feeding. Most farmed crustaceans are benthic, and their feeding behavior is difficult to assess, which is the main difficulty in current research on crustacean feeding. However, behavioral indicators of feeding times, dietary visits, remaining diet and dietary discarding were used to demonstrate that the application of rapeseed meals could also cause anorexia in *L. vannamei*, which will provide insights into the impact of rapeseed meals on crustacean feeding.

Considering the key role of neuropeptides in appetite regulation, it is particularly important to analyze the neuropeptide pathways of rapeseed meal-induced anorexia. Currently, the main methods for analyzing the neuropeptides of crustaceans include mass spectrometry and the transcriptome [18,22]. Based on previous research [26], transcriptome analysis has shown significant advantages over mass spectrometry, such as simple sample preparation, low cost, and easy data analysis [18,22,23]. Therefore, the transcriptome analysis was performed in this study to identify the neuropeptides responsible for anorexia induced by rapeseed meal. Most of the KEGG functional annotations and enriched pathways of transcriptome differential genes were closely related to the regulatory functions of neuropeptides, such as signal transduction, digestive system, sensory system, endocrine system, phototransduction–fly, thyroid hormone signaling pathway and pancreatic secretion. Evidence from KEGG functional annotation and enrichment has shown that rapeseed meal-induced anorexia in *L. vannamei* may be closely related to the regulation of neuropeptides.

To further analyze the neuropeptides related to rapeseed meal-induced anorexia, this study constructed a private database with 430 neuropeptides [26], innovatively introduced the alignment matrix method, and obtained 49 possible neuropeptide transcripts among differentially expressed genes. Although the current method successfully identified potential neuropeptides in differentially expressed genes, the methodological approach could be further optimized. The neuropeptide database should be continuously updated for the species studied. In this study, multiple extremely similar neuropeptides were included in the database; this resulted in multiple neuropeptides being mapped to the same protein and significantly increased the workload for subsequent analysis. However, the establishment of a neuropeptide database has to take into account the extreme similarity between neuropeptide subtypes. Therefore, the neuropeptide database should be optimized based on existing research progress and the purpose of current research.

Under the screening conditions of this study, neuroparsin, orcomyotropin, orcokinin 1, orcokinin 2, bursicon β2, allatostatin B, and YRPRP4 were considered to be involved in the response to rapeseed meal. A recent study on crustaceans confirmed that orcokinin 1, orcokinin 2, allatostatin B, orcomyotropin and YRPRP4 all showed significant changes after feeding, indicating that these neuropeptides are directly involved in appetite regulation [23,30]. Although bursicon β has been characterized in multiple crustaceans, its function in appetite regulation has not been confirmed [31]. Evidence in Drosophila revealed that bursicon β plays an important role in food intake regulation [32], and it can be speculated that bursicon β also plays a similar role in the food intake regulation of *L. vannamei*. Neuroparsin is highly expressed in major appetite-regulating tissues [33], and it has a strong correlation with appetite-regulating insulin-like peptides [34] and the adipokinetic hormone [35]. The above evidence indicates that six of the seven neuropeptides involved in responding to rapeseed meals identified in this study have direct functions in regulating food intake, and the other one was also strongly involved in pathways related to food intake regulation.

Generally speaking, genes that are highly expressed after feeding are involved in the regulation of appetite suppression, while genes that are low expressed after feeding are involved in the regulation of appetite promotion. Studies have confirmed that orcomyotropin and orcokinin are poorly expressed or even not expressed after feeding [36], while allatostatin B is highly expressed [23]. This study showed that after consuming rapeseed meal diet, the expression of genes encoding orcomyotropin and orcokinin increased significantly, while the expression of the gene encoding allatostatin B decreased significantly. The above evidence has shown that the addition of rapeseed meal inhibits the feeding of Litopenaeus vannamei, and the shrimp maintains a strong appetite in preparation for receiving a more palatable diet. However, although we demonstrated that the gene expression of neuroparsin, YRPRP4, and bursicon β2 was reduced in response to rapeseed meal-mediated appetite regulation, the orexigenic functions of these neuropeptides have not yet been elucidated.

## 5. Conclusions

In summary, this study analyzed the anorexia caused by rapeseed meal and the anorexigenic neuroendocrine pathway in *L. vannamei* based on feeding behavior and transcriptomic methods. The results indicated that rapeseed meal caused anorexia in shrimps, as evidenced by the significantly increased remaining diet and dietary discarding, and significantly reduced the dietary visits. The functions involved in rapeseed meal-induced anorexia mainly included signal transduction, the digestive system, sensory system, endocrine system, phototransduction–fly, thyroid hormone signaling pathway and pancreatic secretion. Subsequently, this study identified seven neuropeptides from differential genes, six of which were confirmed to be directly involved in the regulation of food intake and were key neuroendocrine nodes for rapeseed meal-induced anorexia.

## Figures and Tables

**Table 1:** Ingredient formulation and proximate composition of the diets.

Ingredients g·kg^−1^	RSM0	RSM50
Fish meal (67% protein)	150	150
Soybean meal (49.3% protein)	396	198
Peanut meal (49.8% protein)	120	120
Shrimp shell powder (49.7% protein)	50	50
Fermented rapeseed meal (45.1% protein)	0	216
Corn starch	70	70
Methionine	2.6	1.9
Lysine	0	1.2
Fish oil	12	12
Soybean oil	13	16
Soy lecithin	15	15
Cholesterol	5	5
Calcium dihydrogen phosphate	20	20
Composite multidimensional ^1^	20	20
Composite multi-mineral ^2^	20	20
Adhesive CMC	30	30
Choline chloride	5	5
Antioxidant BHT	0.5	0.5
Chromium oxide	0.5	0.5
Betaine	10	10
Cellulose	60.4	38.9
Total	1000	1000
Approximate composition (%)		
Crude protein	35.80	36.71
Crude fat	8.64	8.35
Ash content	11.26	11.69
Moisture	10.54	10.47

^1^ Vitamin premix (g·kg^−1^): thiamin HCl 0.5; riboflavin 3.0; pyridoxine HCl 1.0; DL-calcium pantothenate 5.0; nicotinic acid 5.0; biotin 0.05; folic acid 0.18; vitamin B12 0.002; choline chloride 100.0; inositol 5.0; menadione 2.0; vitamin A acetate (20,000 IU·g^−1^) 5.0; vitamin D3 (400,000 IU·g^−1^) 0.002; DL-alpha-tocopheryl acetate (250 IU·g^−1^) 8; α-cellulose 865.266. ^2^ Trace mineral premix (g·kg^−1^): cobalt chloride 0.004; cupric sulfate pentahydrate 0.250; ferrous sulfate 4.0; magnesium sulfate heptahydrate 28.398; manganous sulfate monohydrate 0.650; potassium iodide 0.067; sodium selenite 0.010; zinc sulfate heptahydrate 13.193; sodium dihydrogenphosphate 15; filler 38.428.

**Table 2:** Encoding of key neuropeptides in response to rapeseed meal.

Gene ID	CDS ID	Neuropeptide	Position
LOC113824994	XM_027377778.1	Neuroparsin	27–99
LOC113819780	XM_027371973.1	Orcomyotropin	78–88
		Orcokinin1	106–118; 121–133; 136–148; 151–163; 166–178; 181–193; 196–208; 211–223; 226–238; 241–253; 256–268; 271–283;
		Orcokinin2	286–296
LOC113827917	Gene.92714	Bursicon β2	86–102
	XM_027380862.1	Bursicon β2	86–102
LOC113811920	XM_027363774.1	Allatostatin B	189–196
	Gene.42028	Allatostatin B	214–221
LOC113811755	XM_027363562.1	YRPRP4	3666–3678
	XM_027363563.1	YRPRP4	3643–3655
	XM_027363561.1	YRPRP4	3678–3690
	XM_027363559.1	YRPRP4	3686–3698
	XM_027363564.1	YRPRP4	3610–3622
	XM_027363560.1	YRPRP4	3679–3691
	Gene.41176	YRPRP4	3465–3477
	Gene.41353	YRPRP4	3498–3510
LOC113811756	XM_027363565.1	YRPRP4	2374–2386

## Data Availability

Data will be made available on request.

## Supplementary Materials

The following supporting information can be downloaded at: https://www.mdpi.com/article/10.3390/ani14040540/s1, Supplementary Material File S1: Analysis results script for neuropeptide comparison; Supplementary Table S1: Prediction results of potential neuropeptides (sub-table 1) and coding information of screened neuropeptides (sub-table 2).
